# Properties of [^18^F]FAPI monitoring of acute radiation pneumonia versus [^18^F]FDG in mouse models

**DOI:** 10.1007/s12149-024-01903-x

**Published:** 2024-02-26

**Authors:** Mingyu Liu, An Yao, Zili Li, Jianping Zhang, Caiyue Ren, Yuyun Sun, Guang Ma, Yun Sun, Jingyi Cheng

**Affiliations:** 1 Department of Nuclear Medicine, Shanghai Proton and Heavy Ion Center, Fudan University Cancer Hospital, Shanghai, 201321 China; 2 Shanghai Key Laboratory of Radiation Oncology (20dz2261000), Shanghai, 201321 China; 3 Shanghai Engineering Research Center of Proton and Heavy Ion Radiation Therapy, Shanghai, 201321 China; 4 Department of Nuclear Medicine, Zhujiang Hospital of Southern Medical University, Guangzhou, 510282 Guangdong Province China; 5 Department of Nuclear Medicine, Shanghai Proton and Heavy Ion Center, Shanghai, 201321 China

**Keywords:** Lung neoplasms, Acute radiation inflammation, Radiation response, [^18^F]FAPI

## Abstract

**Objective:**

In this study, the uptake characteristics of [^18^F]fibroblast activation protein inhibitor (FAPI) molecular imaging probe were investigated in acute radiation pneumonia and lung cancer xenografted mice before and after radiation to assess the future applicability of [^18^F]FAPI positron emission tomography/computed tomography (PET/CT) imaging in early radiotherapy response.

**Methods:**

Initially, the biodistribution of [^18^F]FAPI tracer in vivo were studied in healthy mice at each time-point. A comparison of [^18^F]FAPI and [^18^F]fluorodeoxyglucose (FDG) PET/CT imaging efficacy in normal ICR, LLC tumor-bearing mice was evaluated. A radiation pneumonia model was then investigated using a gamma counter, small animal PET/CT, and autoradiography. The uptake properties of [^18^F]FAPI in lung cancer and acute radiation pneumonia were investigated using autoradiography and PET/CT imaging in mice.

**Results:**

The tumor area was visible in [^18^F]FAPI imaging and the tracer was swiftly eliminated from normal tissues and organs. There was a significant increase of [^18^F]FDG absorption in lung tissue after radiotherapy compared to before radiotherapy, but no significant difference of [^18^F]FAPI uptake under the same condition. Furthermore, both the LLC tumor volume and the expression of FAP-ɑ decreased after thorax irradiation. Correspondingly, there was no notable [^18^F]FAPI uptake after irradiation, but there was an increase of [^18^F]FDG uptake in malignancies and lungs.

**Conclusions:**

The background uptake of [^18^F]FAPI is negligible. Moreover, the uptake of [^18^F]FAPI may not be affected by acute radiation pneumonitis compared to [^18^F]FDG, which may be used to more accurately evaluate early radiotherapy response of lung cancer with acute radiation pneumonia.

**Supplementary Information:**

The online version contains supplementary material available at 10.1007/s12149-024-01903-x.

## Introduction

Over 50% of non-small cell lung cancer (NSCLC) patients receive radiotherapy at least once during curative and palliative treatment [1]. The [^18^F]fluorodeoxyglucose (FDG) uptake in inflammatory tissues encircling the tumor is a limitation of therapy response assessment based on [^18^F]FDG positron emission tomography/computed tomography (PET/CT) [2]. Radiation pneumonitis (RP) is an inflammatory reaction within lung tissue in response to radiation injury and it appears as enhanced [^18^F]FDG uptake [3]. Marks et al. observed that inflammatory alterations in the lungs are noticeable on radiographic images of 30–90% of patients following 30–70 Gy radiotherapy [4]. Researchers have attempted to improve the accuracy of target recognition by employing a suitable threshold and delayed imaging, but they have been unable to address the overlap issue on a fundamental level.

The recent development of a fibroblast activation protein inhibitor (FAPI)-targeted PET/CT molecular probe has yielded encouraging preclinical and clinical results with remarkably high uptake and image contrast [5, 6]. The distinctive benefits of the FAPI probe have garnered the interest of the radiotherapy community. Recent reports indicate that some radiologists have utilized the FAPI probe to delineate tumor targets, including lung cancer [7] and glioblastoma [8]. These studies demonstrated that the FAPI probe has the potential to accurately identify malignancies. We are aware that fibroblasts are normally dormant and become active in response to wound repair. In scars and chronic tissues, activated fibroblasts are also a significant component of the wound-healing response. In chronic fibrosis and tumor fibrosis, fibroblasts exhibit persistent activation, unlike in acute inflammation [9]. In theory, acute inflammation does not result in FAP activation. We pondered whether [^18^F]FAPI is superior to [^18^F]FDG in distinguishing lung cancer tissue from acute RP in light of the results.

Qin et al. [10] monitored the development of lung tissue damage after radiotherapy and determined the uptake of [^18^F]FAPI in the stage of pulmonary fibrosis in a rat model, which is consistent with the results of previous studies. However, no studies have focused on the acute inflammatory stage. Thus, studies on mouse model were conducted as a proof-of-concept and to evaluate the tracer efficacy of [^18^F]FAPI in comparison to [^18^F]FDG.

## Materials and methods

### Preparation of ^18^F-labeled FAPI-04 solutions

FAPI-04 was chosen for this investigation due to its high affinity for FAP. And we automatically synthesized [^18^F]AlF-NOTA-FAPI-04 ([^18^F]FAPI) using a modified procedure based on Siemens’ module. The radiosynthesis of [^18^F]FAPI was started from precursor NOTA-FAPI-04 followed by ^18^F conjugation under optimized reaction conditions according to Kou Y et al. [11]. The final [^18^F]FAPI tracer formulation had a radioactivity yield of 40.3%, radiochemical purity of > 99.9%, and molar activity of 4.59 GBq/mol. The stability of [^18^F]FAPI was checked by analytical HPLC. After 4 h of incubation in human serum at room temperature, the results showed there was no decomposition and no increase in chemical or radiochemical impurities were observed. The stability of [^18^F]FAPI in human serum after 4 h of incubation was > 99%. This stability assures that the drug can be transported and detected in vivo.

### Preparation of acute radiation pneumonia mouse model

For in vivo experiments, 8-week-old ICR mice (Shanghai JIHUI) were used to evaluate the whole-body biodistribution. For the remainder of the in vivo investigation, 8-week-old C57BL/6 mice (Shanghai JIHUI) were used. All animal investigations were conducted in accordance with the guidelines of the Institutional Animal Care and Use Committee (IACUC) (Reference Number: M-2020-002). The protocol was authorized by the Shanghai Proton and Heavy Ion Centre IACUC.

For the acute radiation pneumonia model, O_2_/isoflurane (2% isoflurane, 1.2 L/min O_2_) was used to anesthetize C57BL/6 mice. As the photon beam, a 225 kVp X-ray beam was utilized (PXi precision X-RAD 225, dose rate = 2.042 Gy/min, 225 kV, 13.3 mA, and a source-to-skin distance (SSD) of 50 cm). In addition, a single 15 Gy X-ray dose was administered to the entire thorax (3 cm × 2 cm, the entire lung), while the head, abdomen, and extremities were shielded with lead strips. For the subsequent experiment, an [^18^F]FAPI or [^18^F]FDG tracer was injected into the tail vein of mice 7 days after irradiation.

### Preparation of lung cancer-acute radiation pneumonia mouse model

The LLC cell line was purchased from the Shanghai Cell Bank of the Chinese Academy of Sciences. The cells were grown at 37 °C in 5% carbon dioxide in Dulbecco modified Eagle medium containing 10% fetal calf serum. LLC cell is a cell line derived from the lung of a C57BL mouse with a tumor caused by the implantation of Lewis lung carcinoma.

For in vivo experiments, 2 × 10^6^ LLC cells (100 μL) were injected subcutaneously into the right axillary of C57BL/6 mice. When tumor xenografts reached approximately 10 mm in diameter, a radioactive tracer was injected into the tail vein of tumor-bearing mice. When the tumor measured approximately 6 mm in diameter, the lung cancer-acute radiation pneumonia model was developed. A single 15 Gy dose of X-ray irradiation was delivered to the entire thorax, including the entire lung and tumor (the method was identical to that used in the acute radiation pneumonia model). In the meantime, tumor size changes were measured on the 0th, 1st, 3rd, 5th, and 7th day after treatment. Length (L) and width (W) of the tumor were measured using a vernier caliper, and tumor volume (TV) was calculated using the formula (LW^2^)/2. The change in TV after treatment was denoted as TV/TV_0_, where TV_0_ means the tumor volume before radiation and TV means the tumor volume 7 days after radiation.

### Biodistribution by γ-counter

Normal mice were injected with [^18^F]FAPI (0.74 MBq/mouse) (20 μCi) via the tail vein to evaluate the biodistribution throughout the body. The animals (*n* = 3 for each time point) were euthanized 30 min, 1 h, 1.5 h, and 2 h after injection. For the bone and muscle, a portion of the hind limb was taken. All organ and blood samples were dissected, weighed, and radioactivity was measured with a γ-counter (GC-911, ZONKIA). Radioactivity counts were normalized using an ^18^F standard solution for calibration. The results were displayed as a percentage of the dose injected per gram of tissue (%ID/g). Regarding the acute radiation pneumonia model, the right upper lung (RUL), the right middle lung (RML), the right lower lung (RLL), the left upper lung (LUL), the left lower lung (LLL), and a portion of the left hind limb thigh muscle (M) were removed, and then lung tissue-to-background ratios were calculated.

### In vitro Autoradiography

Autoradiography was used to validate the accuracy of PET/CT data by measuring tracer absorption in lung and tumor tissues. Each mouse was injected with 0.1 mL of 3.7 MBq (100 μCi) [^18^F]FAPI or [^18^F]FDG tracer via the tail vein. Mice were sacrificed under isoflurane-induced anesthesia 1 h after injection. Lungs in the acute radiation pneumonia model and tumors in the lung cancer-acute radiation pneumonia model were dissected and collected, respectively. The dissected samples were immediately made into frozen coronal sections. The sections were tableted on the phosphorimaging plate (BAS-IP TR 2025 E, GE Healthcare) for 24 h and then were read out on the multi-function laser imager (Typhoon FLA 9500, GE Healthcare) by selecting the Phosphor Image scanning mode. The captured data were analyzed and processed with ImageQuant TL version 8.1 software (GE Healthcare).

### Mouse PET/CT imaging

One hour after intravenous injection of [^18^F]FAPI or [^18^F]FDG (3.7 MBq/mouse) (100 μCi), a static PET scan was performed using a small-animal PET scanner (Inveon, Siemens). During the scan, mice were maintained under 2% isoflurane and two-bed positions were acquired (a 5-min CT scan followed by a 10-min PET scan). The PET raw data were reconstructed iteratively using the three-dimensional ordered-subset expectation maximization/maximum algorithm (OSEM3D/MAP, Siemens) and were converted to standard uptake value (SUV) images. To calculate maximal SUV (SUV_max_), ellipsoid sphere regions of interest were manually placed on the tumor of PET images with reference to fused PET/CT images.

### Pathologic evaluations

After the micro-PET scan, anesthesia was used to kill the mice. The lungs of the acute radiation inflammation model were stained with Hematoxylin–eosin (HE). Lungs were removed, perfused, immersed in 4% paraformaldehyde for 24 h, dehydrated via a series of ethanol concentrations, and embedded in paraffin. HE-stained serial 4-μm tissue sections were examined under a light microscope. On the tumor of lung cancer-acute radiation pneumonia model, FAP and glucose transporter (GLUT-1) expression was confirmed by immunofluorescent (IF) staining. Anti-FAP-ɑ primary antibody (PA5-99313, Invitrogen) and anti-GLUT-1 primary antibody (ab115730, abcam) were used to stain FAP-ɑ and GLUT-1 in tumor samples overnight at 4 °C. Next, the Alexa Fluor-conjugated secondary antibodies (DD12, talent biomedical) were added drop by drop, followed by a 50-min incubation at 37 °C. DAPI was then used to counterstain cell nuclei. The section was affixed with an anti-quenching mounting medium before being photographed with a fluorescence microscope.

### Statistical analysis

SPSS 22.0 software was used for the statistical analysis. A two-sided* p* < 0.05 indicated statistical significance. All values were expressed as mean values ± standard deviation. For normally distributed populations, the differences between the two groups were compared using an unpaired *t*-test.

## Results

### Normal-organ biodistribution and tumor uptake of [^18^F]FAPI

**Fig. 1:**
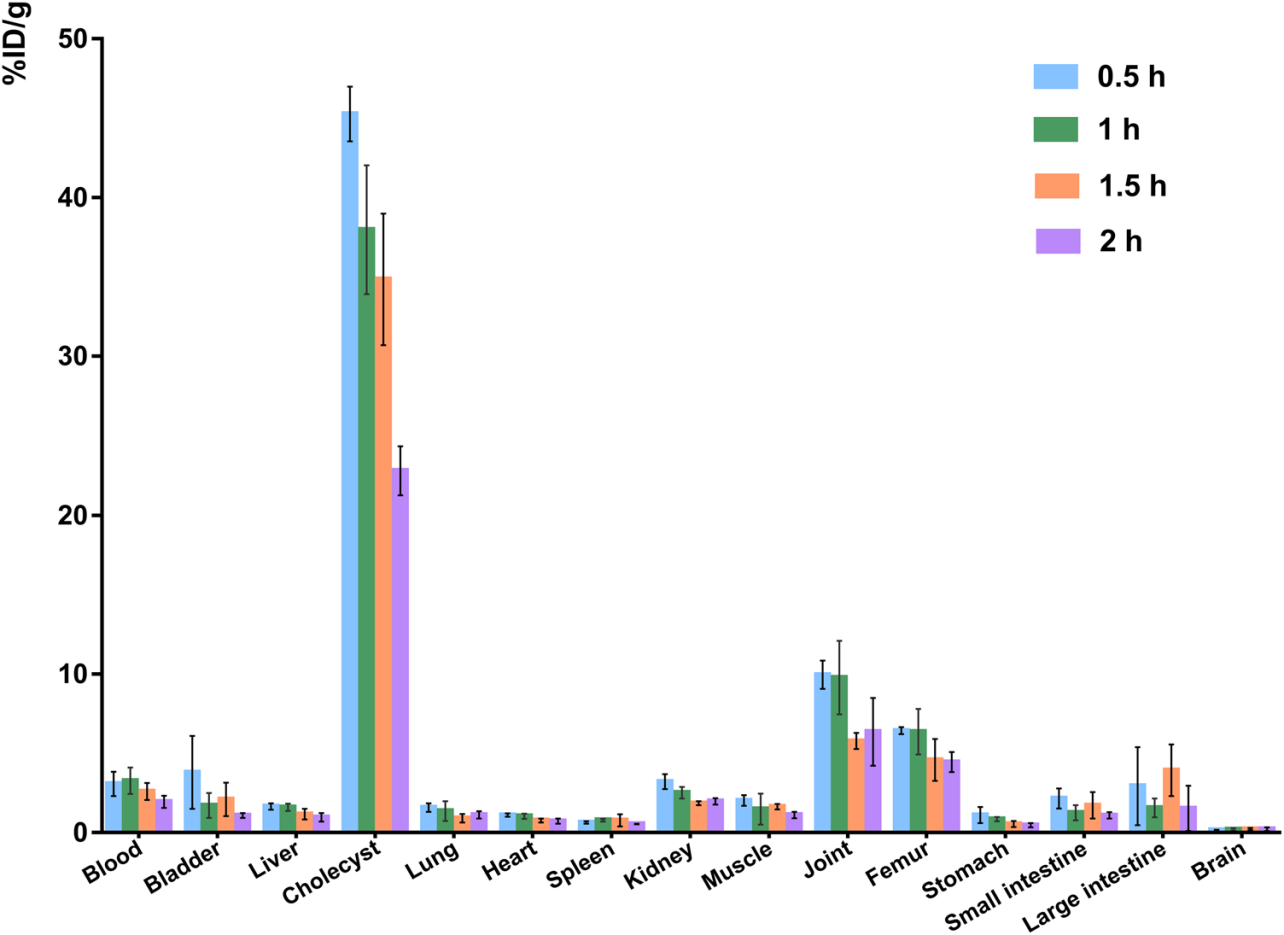
Time-dependent bio-distribution of [^18^F]FAPI-04 in normal mouse organs at 0.5 h, 1 h, 1.5 h, and 2 h after intravenous injection via tail vein

**Fig. 2:**
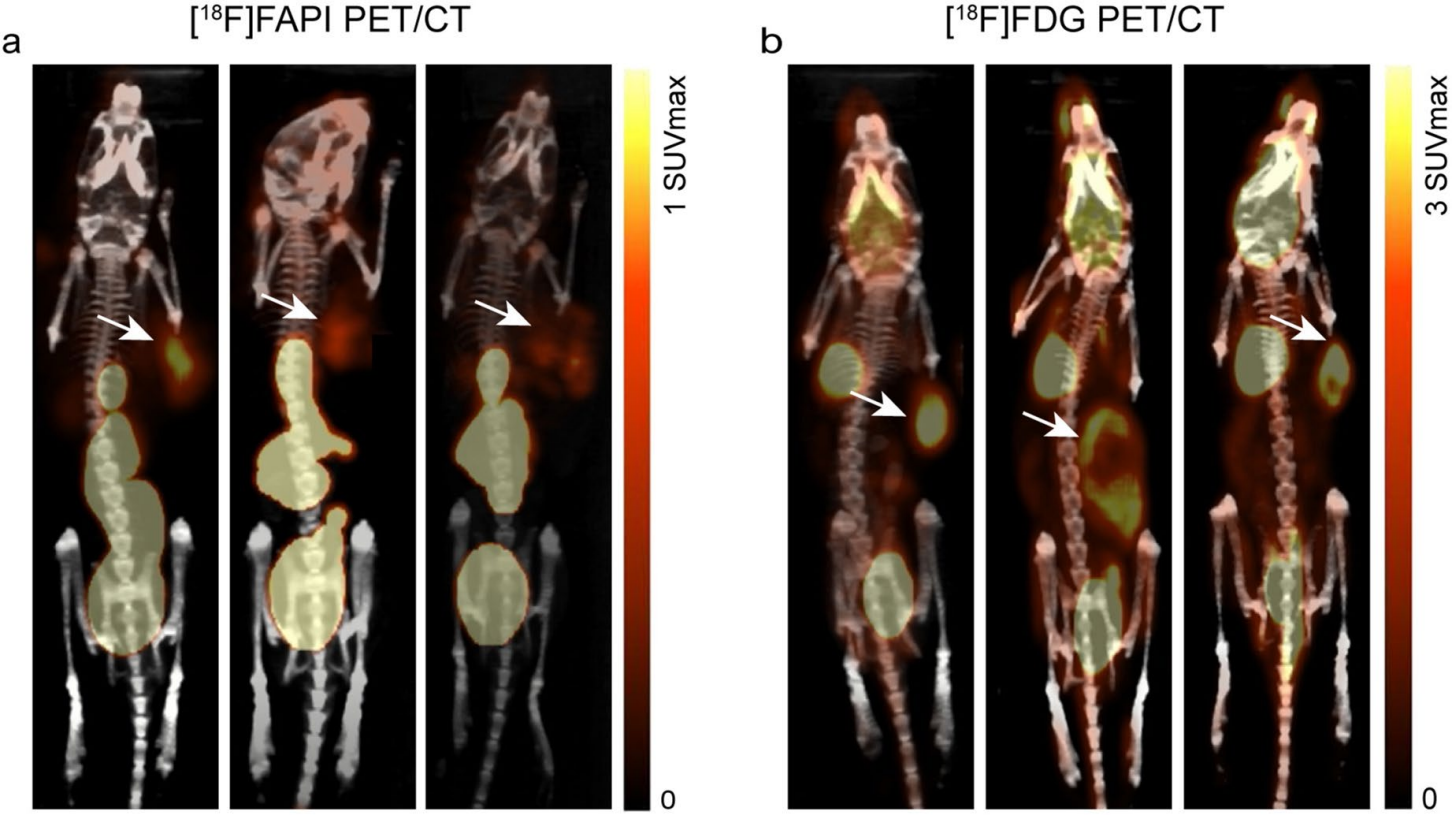
Comparison of uptake rates between [^18^F]FAPI-04 (1 h after injection) and [^18^F]FDG (1 h after injection) PET imaging in LLC xenograft model. (Arrows indicate tumor xenograft)

After intravenous injection of [^18^F]FAPI solution, the organs of some mice were resected and the radioactivity was monitored by a gamma counter. The biodistribution data showed pronounced %ID/g uptake of [^18^F]FAPI in the cholecyst, weak signals in joints and bones, and low signals in the key organs such as the heart and brain. And 2 h after i.v. injection, the [^18^F]FAPI probe was excreted gradually in the tumor-prone areas such as the liver, lung, bone, brain and other normal tissues and organs, except the cholecyst (Fig. 1). In vivo imaging by small animal PET/CT further confirmed the high accumulation of [^18^F]FAPI in tumors with scarcely signal in the bone which showed the good in vivo radio stability of [^18^F]FAPI (Fig. 2). Unexpectedly, high and specific uptake of [^18^F]FDG was detected in bone structures, liver and brain. However, gamma counter and PET/CT results of [^18^F]FAPI revealed that scarce uptake was monitored in the liver, heart, brain, and other critical organs. Only joint and bone showed weak signals. This is quite different from the [^18^F]FDG characteristics. [^18^F]FAPI provides a good pharmacokinetics in xenografts and opens a door for FDG-insensitive tumors.

### In vivo distribution of [^18^F]FAPI and [^18^F]FDG in C57/BL mice after radiotherapy

**Fig. 3:**
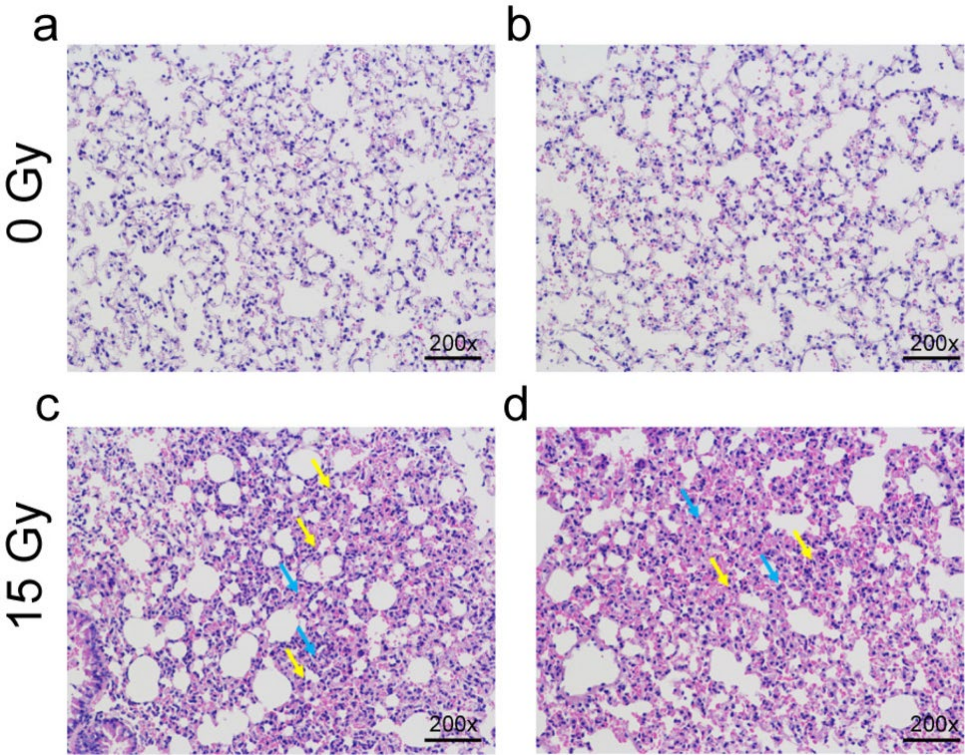
Radiation xenograft model of acute pneumonia. Hematoxylin–eosin (HE) staining of normal (**a** and **b**) and acute radiation (**c** and **d**) lung sections in C57BL/6 xenograft model. The yellow arrow indicates neutrophils and mononuclear cell infiltration, and the blue arrow indicates epithelial hyperplasia

**Fig. 4:**
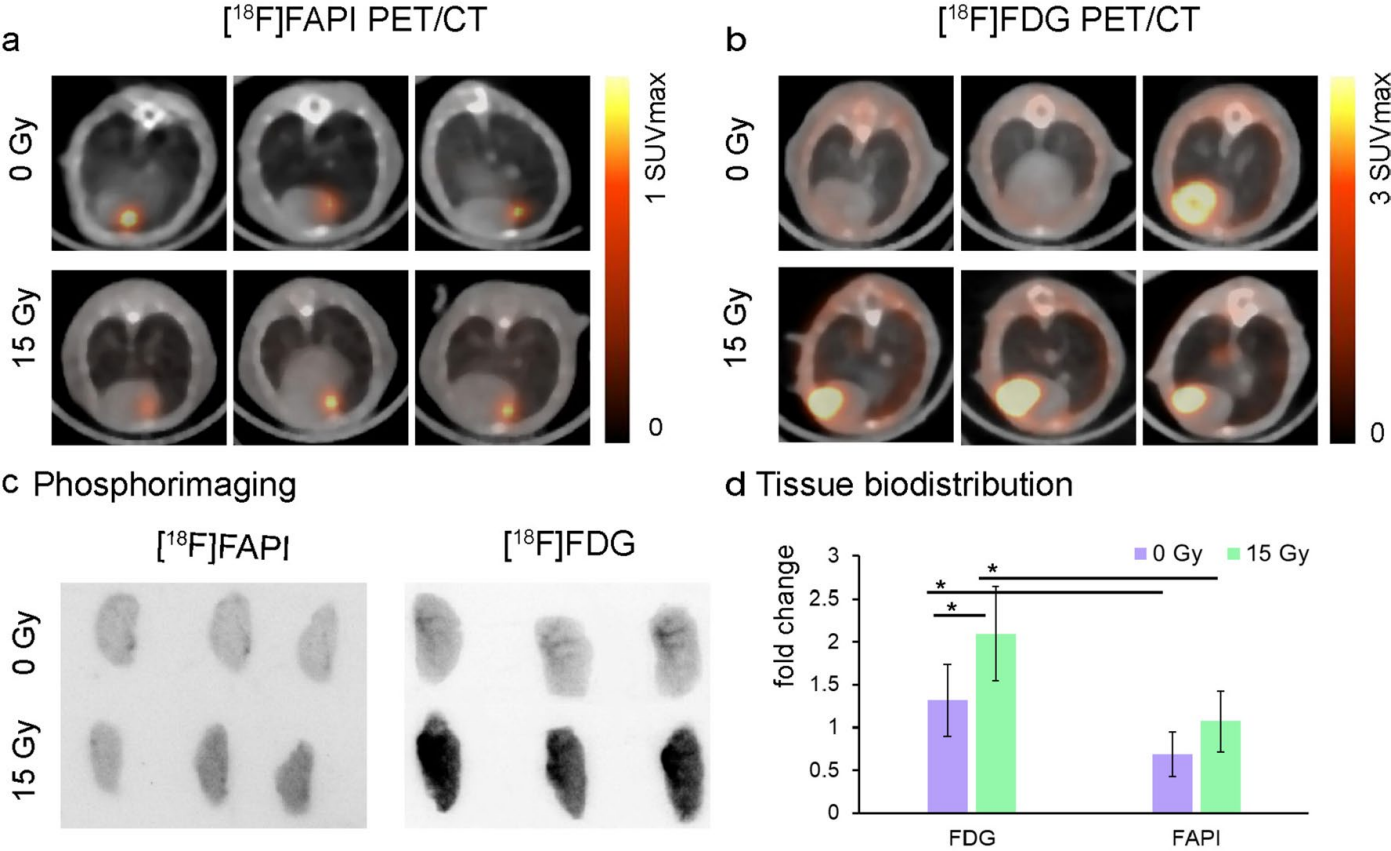
Tracer uptake in normal and acute radiation pneumonia xenograft model on [^18^F]FAPI (**a**) (1 h after injection) and [^18^F]FDG (**b**) (1 h after injection). **c** Representative autoradiography of normal and acute radiation lung sections in C57BL/6 xenograft model. **d** Representative bio-distribution of lung tissue (RUL/M) after intravenous administration of [^18^F]FAPI (1 h after injection) or [^18^F]FDG (1 h after injection) in normal and acute radiation pneumonia xenograft model. ^*^P < 0.05. Values are presented as mean ± SD (*n* = 3). The right upper lung (RUL), the left hind limb thigh muscle (M)

HE staining of lung tissues was conducted to confirm the presence of acute radiation pneumonia. The results of the unirradiated group showed that there was no obvious abnormality in pulmonary parenchyma and interstitium. However, the results of the irradiated group showed that the epithelial cells increased, scattered neutrophils and mononuclear infiltration were seen, and no obvious fibrosis was found, indicating that the acute pneumonia model was successfully established (Fig. 3). The biodistribution of [^18^F]FAPI and [^18^F]FDG in irradiated lung tissues were compared (Table 1). The results showed that the uptake rates of [^18^F]FAPI in RUL/M, RML/M, RLL/M, LUL/M and LLL/M were all lower than that of [^18^F]FDG, and the difference was statistically significant (*P* = 0.020, 0.016, 0.016, 0.007, and 0.006, respectively). Similarly, as in the table of the biodistribution studies, figures better discriminated the significantly lower uptake ratios of [^18^F]FAPI compared with [^18^F]FDG (Supplemental Data 1). Autoradiography results confirmed that [^18^F]FDG uptake in lung tissue increased significantly after radiotherapy, but there was no remarkable difference in [^18^F]FAPI uptake in lung tissue before and after radiotherapy. The same result has also been verified in the cross-section of small animal PET/CT imaging (Fig. 4). In summary, the uptake of [^18^F]FAPI in normal lung tissue was lower than that of [^18^F]FDG, which remained the same 7 days after radiotherapy.

**Table 1:** Bio-distribution after intravenous administration of [^18^F]FAPI-04 or [^18^F]FDG in acute radiation pneumonia xenograft model

Site	[^18^F]FDG	[^18^F]FAPI	*t* value	*P* value
RUL/M	2.09 ± 0.55	1.07 ± 0.35	3.145	0.020^*^
RML/M	2.12 ± 0.52	0.98 ± 0.45	3.307	0.016^*^
RLL/M	2.03 ± 0.39	0.89 ± 0.56	3.307	0.016^*^
LUL/M	2.05 ± 0.43	0.83 ± 0.43	4.009	0.007^**^
LLL/M	2.10 ± 0.40	0.93 ± 0.40	4.107	0.006^**^
L/M	2.07 ± 0.40	0.95 ± 0.60	3.084	0.022^*^

*RUL* right upper Lobe, *RML* right middle lobe, *RLL* right lower lobe, *LUL* left upper lobe, *LLL* left lower lobe, *L/M* lung/muscle

Data shown are mean ± SD and result from a pooling of 3 independent experiments

^*^P < 0.05%

^**^P < 0.01

### Comparison of [^18^F]FAPI and [^18^F]FDG in LLC xenografted mouse after radiotherapy

**Fig. 5:**
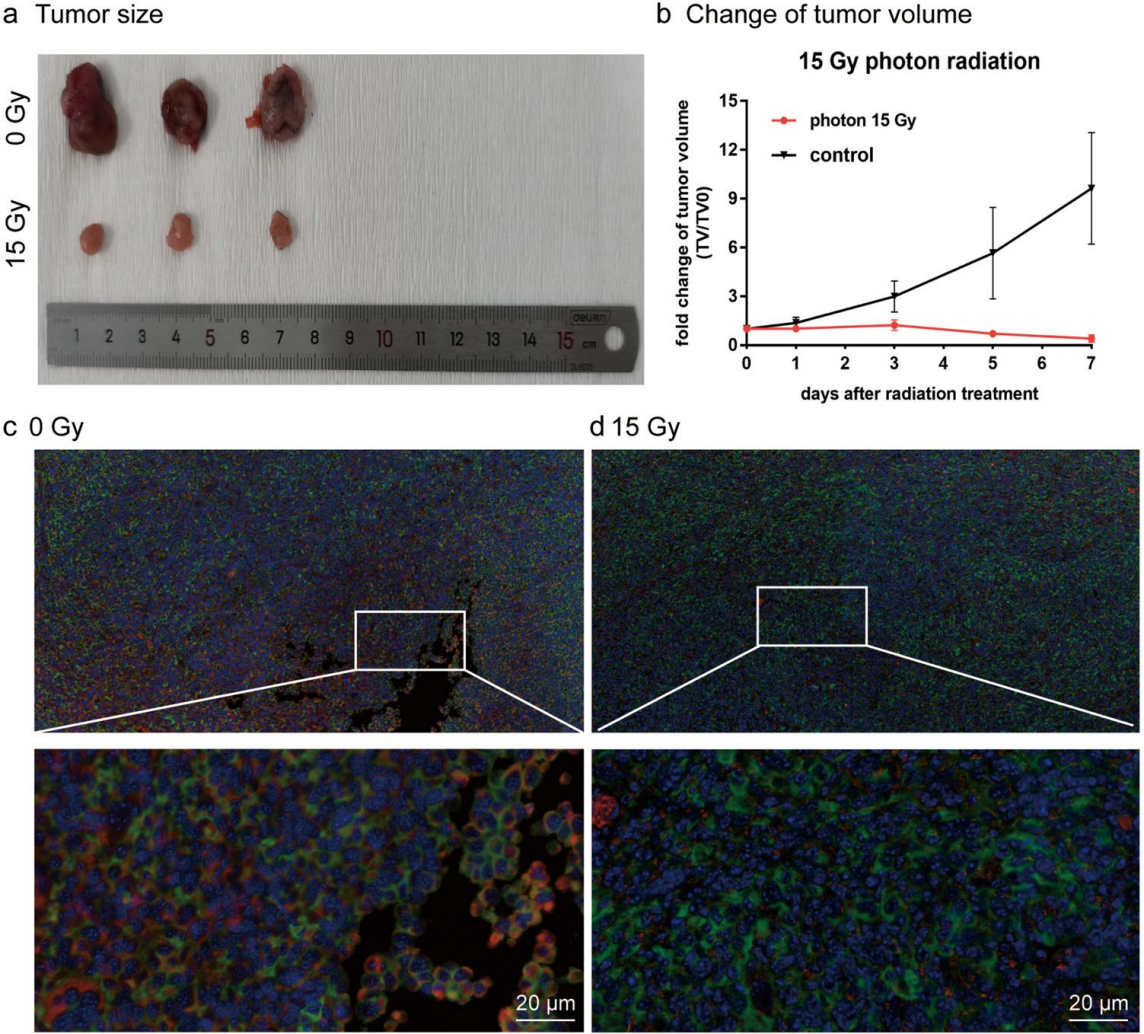
**a** Photograph of resected tumors from each group 7 days after radiotherapy (*n* = 3). **b** Changes in LLC xenografted tumor volume after 15 Gy photon radiotherapy. Data are presented as mean ± SD (*n* = 3). Immunofluorescent staining of LLC tumor xenografts (**c** and **d**) using FAP-α (red) and GLUT-1 (green) antibody (note that blue is DAPI staining of nuclei)

To compare the in vivo targeting properties and pharmacokinetics of [^18^F]FAPI, autoradiography and micro-PET studies in LLC xenotransplant mice were performed. The changes of tumor size were measured on the 0th, 1st, 3rd, 5th and 7th day after intervention in the unirradiated group and irradiated group (a single 15 Gy X-ray irradiation dose). The tumor volume of the unirradiated group was 22.5 ± 9.6 mm^3^, 31.2 ± 16.4 mm^3^, 65.3 ± 26.0 mm^3^, 125.3 ± 66.8 mm^3^ and 222.9 ± 143.6 mm^3^ on the 0th, 1st, 3rd, 5th and 7th day, respectively. The growth rate of tumor volume was 963.1%. At the same time, the tumor volume in the irradiated group was 52.3 ± 14.3 mm^3^, 53.4 ± 14.9 mm^3^, 65.5 ± 26.6 mm^3^, 34.9 ± 10.4 mm^3^ and 23.3 ± 13.1 mm^3^ on the 0th, 1st, 3rd, 5th and 7th day respectively, and the change rate of tumor volume decreased to 41.2% on the 7th day (Fig. 5b). Compared with the unirradiated group, the tumor volume of the irradiated group decreased and the tumor proliferation was inhibited 7 days after radiation (Fig. 5a). Then, LLC tumors were dissected and IF staining was performed to observe the expression of FAP-α and GLUT-1. The staining showed that both FAP-α and GLUT-1 proteins were positive in the unirradiated group (Fig. 5c). However, the intensity and activity of FAP-α in the irradiated group were weaker than in the unirradiated group, and there was no significant change in GLUT-1 expression compared with the unirradiated group (Fig. 5d) (Supplemental Data 2).

**Fig. 6:**
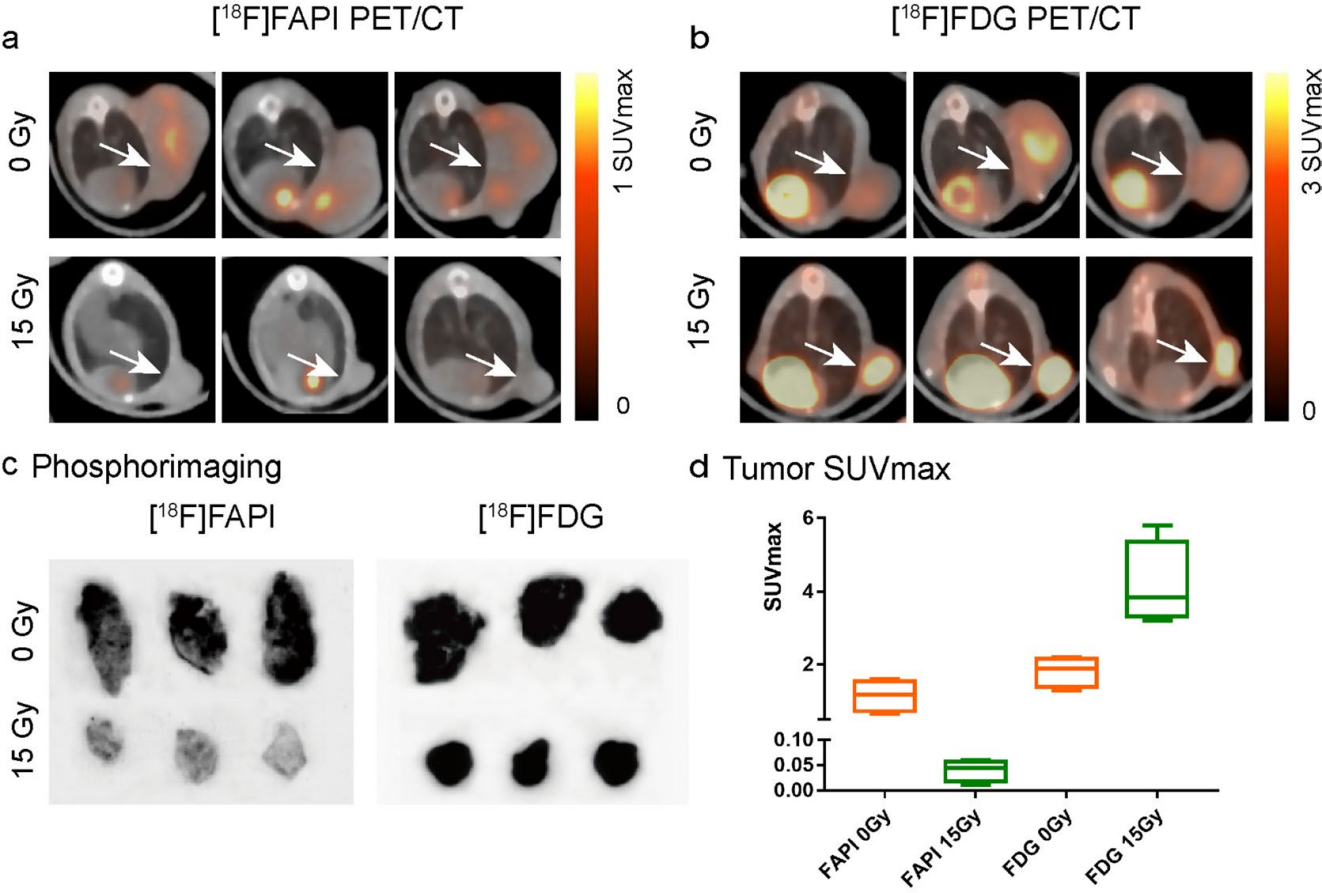
Tracer uptake in LLC tumor of normal and radiation-treated mice on [^18^F]FAPI (**a**) (1 h after injection) and [^18^F]FDG (**b**) (1 h after injection). **c** Representative autoradiography of LLC tumor in normal and radiation-treated mice. **d** SUV_max_ of LLC tumor measured on PET imaging

After intravenous injection of [^18^F]FAPI and [^18^F]FDG solution, one group of mice were monitored by a small animal PET/CT. In the unirradiated group, apparent uptake of [^18^F]FAPI and [^18^F]FDG in LLC tumors and faint uptake in lungs were found. Seven days after radiation, [^18^F]FAPI PET/CT imaging showed that the tumor proliferation was inhibited and showed a weak [^18^F]FAPI signal in LLC tumors and lungs, which was consistent with FAP-α staining in LLC tumors. However, [^18^F]FDG PET/CT imaging showed that the tumor proliferation was inhibited but still showed a positive [^18^F]FDG signal in LLC tumors and lungs, which was consistent with GLUT-1 staining in LLC tumors (Fig. 6a, b). Meanwhile, uptake rates were compared between [^18^F]FAPI and [^18^F]FDG (Fig. 6d). It was found that SUV_max_ uptake of [^18^F]FAPI in the irradiated group (SUV_max_ = 0.04 ± 0.02) was lower than that in the unirradiated group (SUV_max_ = 1.15 ± 0.43), and the difference was statistically significant (*t* = 5.143, *P* = 0.014). As for [^18^F]FDG, we found that the SUV_max_ uptake in the unirradiated group was 1.83 ± 0.41, and the uptake in the irradiated group was 4.18 ± 1.13, which was significantly higher than that in the unirradiated group (*t* = 3.901, *P* = 0.008). Autoradiographic images also showed heterogeneously positive [^18^F]FAPI signal and deep [^18^F]FDG signal in the unirradiated group, but shallow [^18^F]FAPI signal and strong [^18^F]FDG signal still in the irradiated group (Fig. 6c).

## Discussion

Fifteen to forty percent of patients receiving radiation therapy for NSCLC develop a severe dose-limiting complication called RP [12]. [^18^F]FDG uptake is not tumor-specific, as it also occurs in areas of increased cellular metabolism caused by inflammation [13]. Early response assessment functions as an early predictor of treatment outcome and enables therapy to be modified to maximize its benefit. However, the problem of inflammatory alterations in normal tissue during and after radiotherapy hinders [^18^F]FDG PET/CT-based early assessment of radiotherapy response [14]. The purpose of this investigation was to determine whether [^18^F]FAPI can accurately identify tumors, independent of acute radiation-induced lung inflammation.

This is the first report comparing the characteristics of [^18^F]FAPI and [^18^F]FDG uptake in acute radiation inflammation. In our feasibility investigation, we investigated the tumor uptake and distribution of the [^18^F]FAPI probe in normal organs. [^18^F]FAPI PET/CT offers high tumor-to-background contrast ratios and does not require a special diet or fasting before the examination. In addition, the results demonstrated a low absorption of [^18^F]FAPI in tumor-prone tissues such as the liver, lung, bone, and brain, which opens the door for [^18^F]FDG-resistant tumors. This is analogous to the findings of Frederik et al. [15], who discovered that the background uptake in the brain and liver was significantly lower with [^68^Ga]Ga-FAPI.

HE staining of the irradiated group in this analysis revealed increased epithelial cells, scattered neutrophils, and mononuclear infiltration. The histological examination of the irradiated rodents confirmed the presence of pathological changes associated with acute RP. After radiation exposure, damaged lung cells release cytokines that recruit inflammatory cells to the alveoli and interstitium, causing acute-phase pneumonia [16]. Experiments using a model of acute radiation pneumonia revealed that [^18^F]FDG uptake in lung tissue increased substantially after radiotherapy, whereas there was no significant difference between [^18^F]FAPI uptake before and after radiotherapy. The inflammatory response can induce the Warburg effect [17], similar to malignant tumors. Thus, we concluded that the increased absorption of [^18^F]FDG in lung tissue was caused by the Warburg effect of inflammatory cells. Importantly, we initially detected minimal [^18^F]FAPI uptake in the lung tissue of irradiated rats. This result was consistent with a previous study by Schmidkonz et al. which demonstrated the feasibility of the [^68^Ga]Ga-FAPI PET/CT probe for differentiating between inflammatory and fibrotic activities in 27 patients with IgG_4_-related disease [18]. Fibroblasts are persistently activated in chronic fibrosis and tumor fibrosis, but not in acute inflammation [9]. In the study by Qin et al. [10], [^18^F]FAPI uptake was observed on day 7 in the 90 Gy group with foamy macrophages and slight collagen deposition present which should be classified as chronic inflammatory phase. The reason for the inconsistency with our results may be the difference in radiation dose and the radiosensitivity of different murine.

Our small animal PET investigations utilizing LLC xenografts revealed increased [^18^F]FDG uptake in the radiation-treated tumors and lungs, while [^18^F]FAPI uptake was weak. Moreover, autoradiographic images and protein expression variations in the tumor were consistent with PET images. Overall, these results indicated that acute RP may not influence the uptake of [^18^F]FAPI compared to [^18^F]FDG. [^18^F]FAPI may be able to evaluate the tumor’s early radiotherapy response more precisely than [^18^F]FDG, according to the results. This comparison also obviously demonstrated the superiority of [^18^F]FAPI for monitoring certain [^18^F]FDG-resistant lesions.

Our research has several limitations. One limitation is that we established the model using a single high-dose irradiation which differs marginally from clinical practice. Another limitation is the small number of animals used in this investigation; our method should be validated with a larger sample size. Our findings regarding [^18^F]FAPI uptake in the lungs may not accurately reflect the human condition, as we only examined rodents and not human patients.

## Conclusion

There is no defluorination of [^18^F]FAPI in vivo, and its background uptake is negligible. [^18^F]FAPI may have the advantage of detecting [^18^F]FDG-insensitive lesions, as the uptake of [^18^F]FAPI may not be affected by acute RP compared to [^18^F]FDG, which may be used to more accurately evaluate the radiotherapy response of lung cancer with acute radiation pneumonia.

### Supplementary Information

Below is the link to the electronic supplementary material.

Supplementary file1 (DOCX 182 KB)

## Data Availability

The datasets used and analyzed during the current study are available from the corresponding author on reasonable request.
