# Supplementary material for: Properties of [18F]FAPI monitoring of acute radiation pneumonia versus [18F]FDG in mouse models
Source: Ann Nucl Med. 2024 Feb 26;38(5):360–8. doi: 10.1007/s12149-024-01903-x (PMC11016509; doi:10.1007/s12149-024-01903-x)
Supplement: Supplementary file 1 — Supplementary file1 (DOCX 182 KB) [file 12149_2024_1903_MOESM1_ESM.docx]

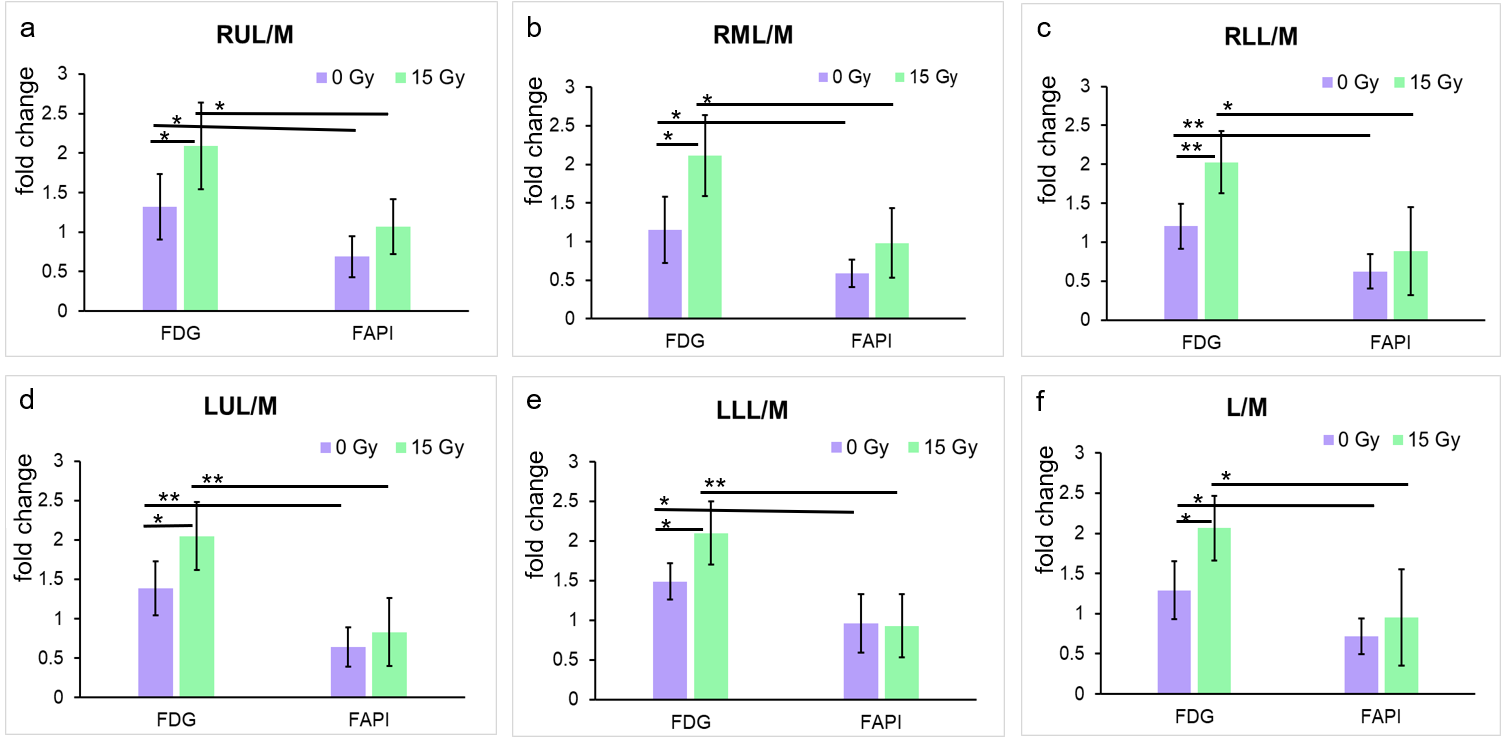
**Supplemental Data 1** Bio-distribution after intravenous administration of [^18^F]FAPI-04 (1 h after injection) or [^18^F]FDG (1 h after injection) in normal and acute radiation pneumonia xenograft model. Data shown are mean ± SD and result from a pooling of 3 independent experiments.


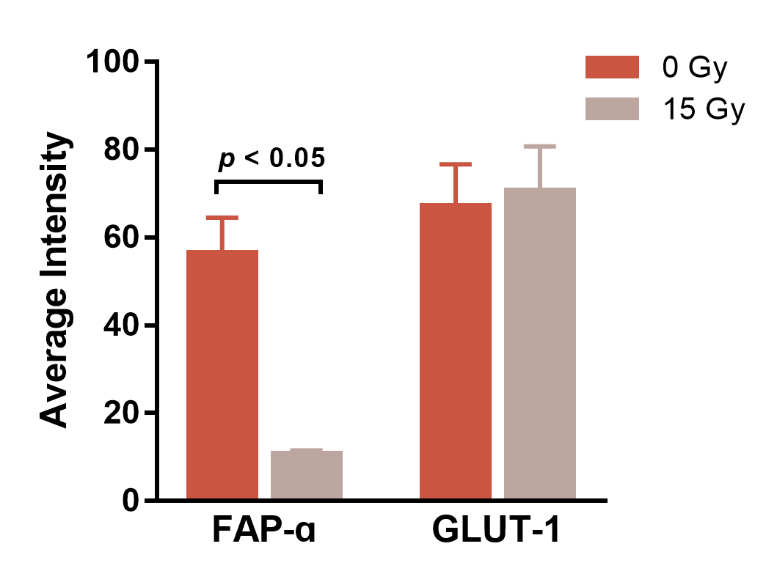


**Supplemental Data 2** Comparison of average intensities of FAP- α and GLUT-1 in immunofluorescence images between the irradiated group and the unirradiated group (n = 3).
